# Jet Injection of Naked mRNA Encoding the RBD of the SARS-CoV-2 Spike Protein Induces a High Level of a Specific Immune Response in Mice

**DOI:** 10.3390/vaccines13010065

**Published:** 2025-01-13

**Authors:** Denis N. Kisakov, Larisa I. Karpenko, Lyubov A. Kisakova, Sergey V. Sharabrin, Mariya B. Borgoyakova, Ekaterina V. Starostina, Oleg S. Taranov, Elena K. Ivleva, Oleg V. Pyankov, Anna V. Zaykovskaya, Elena V. Dmitrienko, Vladimir A. Yakovlev, Elena V. Tigeeva, Irina Alekseevna Bauer, Svetlana I. Krasnikova, Nadezhda B. Rudometova, Andrey P. Rudometov, Artemiy A. Sergeev, Alexander A. Ilyichev

**Affiliations:** 1 State Research Center of Virology and Biotechnology “Vector”, Rospotrebnadzor, World-Class Genomic Research Center for Biological Safety and Technological Independence, Federal Scientific and Technical Program on the Development of Genetic Technologies, 630559 Koltsovo, Russia; lkarpenko1@yandex.ru (L.I.K.); orlova.lyub1996@yandex.ru (L.A.K.); sharabrin_sv@vector.nsc.ru (S.V.S.); borgoyakova_mb@vector.nsc.ru (M.B.B.); starostina_ev@vector.nsc.ru (E.V.S.); taranov@vector.nsc.ru (O.S.T.); ivleva_ek@vector.nsc.ru (E.K.I.); pyankov_ov@vector.nsc.ru (O.V.P.); zaykovskaya_av@vector.nsc.ru (A.V.Z.); yakovlev_va@vector.nsc.ru (V.A.Y.); tigeeva_ev@vector.nsc.ru (E.V.T.); sveta.krasnikova2000@yandex.ru (S.I.K.); andreeva_nb@vector.nsc.ru (N.B.R.); rudometov_ap@vector.nsc.ru (A.P.R.); sergeev_aa@vector.nsc.ru (A.A.S.); ilyichev@vector.nsc.ru (A.A.I.); 2 Institute of Chemical Biology and Fundamental Medicine, Siberian Branch, Russian Academy of Sciences, 630090 Novosibirsk, Russia; elenad@niboch.nsc.ru (E.V.D.); i.bauer@g.nsu.ru (I.A.B.)

**Keywords:** mRNA vaccines, needle-free jet injection, lipid nanoparticles, RBD, SARS-CoV-2, immune response, protective properties

## Abstract

**Background:** Although mRNA vaccines encapsulated in lipid nanoparticles (LNPs) have demonstrated a safety profile with minimal serious adverse events in clinical trials, there is opportunity to further reduce mRNA reactogenicity. The development of naked mRNA vaccines could improve vaccine tolerability. Naked nucleic acid delivery using the jet injection method may be a solution. **Methods:** In the first part of the study, the optimal conditions providing low traumatization and high expression of the model mRNA-GFP molecule in the tissues of laboratory animals were determined. Then, we used the selected protocol to immunize BALB/c mice with mRNA-RBD encoding the SARS-CoV-2 receptor-binding domain (RBD). It was demonstrated that mice vaccinated with naked mRNA-RBD developed a high level of specific antibodies with virus-neutralizing activity. The vaccine also induced a strong RBD-specific T-cell response and reduced the viral load in the lungs of the animals after infection with the SARS-CoV-2 virus. The level of immune response in mice immunized with mRNA-RBD using a spring-loaded jet injector was comparable to that in animals immunized with mRNA-RBD encapsulated in LNPs. **Results:** In this study, the efficacy of an inexpensive, simple, and safe method of mRNA delivery using a spring-loaded jet injector was evaluated and validated. **Conclusions:** Our findings suggest that the jet injection method may be a possible alternative to LNPs for delivering mRNA vaccines against SARS-CoV-2 infection.

## 1. Introduction

Over the past decade, mRNA vaccine technology has proven to be a safe and effective vaccination tool, offering advantages over traditional vaccines [1,2,3]. Numerous mRNA vaccines are currently in clinical trials, underscoring the high level of interest in this technology [4,5,6,7,8,9,10]. For mRNA to function effectively, it must remain intact, undergo minimal extracellular degradation, and successfully penetrate immunocompetent cells. Due to the poor cellular uptake of free nucleic acids, various mRNA delivery methods have been developed using both viral and non-viral systems [11,12]. Non-viral delivery systems use approaches that can be categorized into chemical and physical methods. Among chemical methods, lipid nanoparticle (LNP) delivery is well known; LNPs were used to deliver the first approved mRNA vaccines against COVID-19 [13,14] and provided high efficacy. However, LNP delivery vehicles have inherent immunostimulatory properties and may induce some adverse events such as allergic reactions, renal pathologies, pericarditis, myocarditis, and others [15,16,17]. The development of improved, yet simple and safe, delivery methods could help address the current challenges associated with mRNA-based vaccines [18].

Another approach for mRNA delivery involves physical methods such as electroporation, gene gun, ultrasound, and needle-free jet injection. Among these, needle-free jet injection shows considerable promise as a method for mRNA vaccine delivery [19,20]. This method works by using the kinetic energy of a high-speed fluid jet, generated under pressure through the nozzle’s orifice, to penetrate the dermal layers and deliver pharmaceutical agents into the subcutaneous and intramuscular layers [21]. During injection, changes in shear stress cause the cell membrane to twist, creating temporary pores that facilitate nucleic acid entry into the cell, thereby significantly enhancing the immunogenicity of mRNA vaccines. Unlike traditional intramuscular injection with a syringe, where the needle directly penetrates the skin and delivers a large fluid volume, jet injection disperses an mRNA vaccine more evenly. This dispersion increases the contact area between the injected material and cells [22,23,24,25,26,27,28].

Currently, active research is underway to develop devices capable of delivering mRNA vaccines via jet injection. While several technical solutions exist, jet injectors such as PYRO injectors and spring injectors are currently available on the global market, with each differing in how they generate the pressure needed for drug injection. The PYRO injector uses the chemical energy of an explosive reaction. Due to the difficulty of controlling this reaction, these devices are more complex in design and may offer less precision in the volume of drug delivered. This type of injector also requires additional safety precautions due to the use of explosives [29,30]. In contrast, spring injectors operate using kinetic energy generated by springs. These devices are comparatively simpler, more reliable, safer, and easier to maintain, with better control and reproducibility in drug injection [29,31]. These characteristics make spring injectors the preferred choice for mRNA vaccine delivery. Currently, there are no universal standardized protocols for jet injection. To achieve high vaccine immunogenicity across different animal species when studying jet injection methods, it is essential to consider factors such as weight, skin thickness, and hair cover to optimize injection protocols with respect to jet pressure, flow rate, and injection time.

Previously, we constructed an mRNA encoding the receptor-binding domain of the SARS-CoV-2 S protein (mRNA-RBD) [32]. The receptor-binding domain (RBD) was chosen as an immunogen due to its important roles in interacting with cellular receptors and as a primary target for virus-neutralizing antibodies that prevent the virus from entering cells [33,34,35,36,37,38,39]. Some researchers believe that it is safer to use the RBD domain of the S protein, since the full-length S protein is known to contain fragments that can cause antibody-dependent enhancement of infection [33,34,35,36,37,38,40]. A polyglucin:spermidine conjugate was previously studied as an mRNA-RBD carrier [32]. Next, we decided to explore other alternative methods of delivering the mRNA vaccine in order to increase its delivery efficiency. We proposed jet injection as an efficient, simple method of delivery for mRNA vaccines in this study.

The aim of this study was to determine the optimal jet injection parameters for immunizing BALB/c mice with mRNA-based constructs. In the first part of the study, the optimal conditions providing low traumatization and high expression of the model mRNA-GFP molecule in the tissues of laboratory animals were determined. Then, we used the selected protocol to immunize BALB/c mice with mRNA-RBD encoding the SARS-CoV-2 receptor-binding domain (RBD).

## 2. Materials and Methods

### 2.1. In Vitro mRNA Synthesis

Two genetic constructs were used for mRNA synthesis: pVAX-C1-RBD, encoding the genetic sequence of the S protein SARS-CoV-2 (GenBank MN908947), namely, a fragment of the receptor binding domain (RBD) of protein S (320V-542N), and pVAX-C1-GFP, encoding the sequence of the green fluorescent protein (GFP) gene, which was previously obtained from the Bioengineering Department of the FBRI SRC Vector Rospotrebnadzor. The gene encoding the RBD contained a signal peptide to ensure protein secretion from the cell [41]. The target RBD and GFP genes within the DNA matrix were flanked by 5′ and 3′ NTOs of human α-globin, with a poly(A) tail of 100 nucleotides inserted after the 3′ NTO. Prior to synthesis, the plasmid DNA was linearized through the Bso31I restriction site. mRNA synthesis was performed using an in vitro mRNA synthesis kit (BioLabMix, Novosibirsk, Russia) according to the manufacturer’s protocol with minor modifications: uridine was replaced by N1-methylpseudouridine (BioLabMix, Novosibirsk, Russia). After mRNA synthesis, 1 μL DNase I (1 unit of activity/μL) was added to the reaction mixture and incubated at 37 °C for 30 min to degrade the DNA matrix. The absence of a DNA matrix was confirmed by electrophoretic separation of transcription products in an agarose gel. The synthesized mRNA was purified and isolated from the reaction mixture by precipitation of the product with 5M ammonium acetate followed by washing with 70% ethanol. The mRNA precipitate was dried at room temperature and dissolved in sterile water, and its concentration was measured. To remove dsRNA contaminants, the mRNA was further purified on cellulose in a buffer containing ethanol, as described by Baiersdörfer [42]. RNase inhibitors were not used in the mRNA preparations.

To evaluate the distribution of mRNA vaccine molecules following injection, mRNA-RBD was synthesized. A Cyanine 5 (Cy5) fluorescent tag was incorporated into the 3’ end of the synthesized mRNA using the Cy5 Fluorescent Tag Insertion Kit (BioLabMix, Novosibirsk, Russia). The tag was affixed in accordance with the instructions provided by the manufacturer. In summary, the 3’ end of the mRNA was oxidized for one hour in the dark at room temperature. The preparation was then purified by alcohol precipitation at −20 °C, and the resulting precipitate was dissolved in DEPC-treated water. The Cy5 dye solution was added to the purified oxidized RNA and incubated overnight in a dark place at room temperature. Finally, the purification was repeated by precipitation in an alcoholic solution.

### 2.2. LNP Package and Characterization

Lipid nanoparticles (LNPs) loaded with mRNA-RBD (mRNA-RBD-LNPs) were prepared by mixing an aqueous phase containing mRNA-RBD with an ethanol phase containing a mixture of lipids in a volume ratio of 3:1 using an automated nanoparticle system (ANP System, Dolomite, Royston, UK) involving sequential connection of mixing devices: a Micromixer and a T-chip (Dolomite, Royston, UK). mRNA-RBD was dissolved in citrate buffer (100 mM, pH 4.0). Lipid mixtures were dissolved in anhydrous ethanol at a molar ratio of 50:10:38.5:1.5 according to Moderna’s formulation for ionizable lipid (SM-102, AVT, China, Shenzhen), 1,2-distearoyl-sn-glycero-3-phosphocholine (DSPC, AVT, China, Shenzhen), cholesterol (CDH, New Delhi, India) and PEGylated lipid (DMG-PEG-2000, AVT, China, Shenzhen). The N:P ratio was maintained at 6:1. After the obtained nanoparticles were dialyzed against PBS buffer (pH 7.4) for 24 h, the mRNA-RBD-LNPs were then sterilized using a 0.22 μm filter and stored at 4 °C for further use.

The hydrodynamic particle size of mRNA-RBD-LNPs was determined by dynamic light scattering and measured using the intensity-averaged particle size (Z-average), polydispersity index, and zeta potential using a Zetasizer NanoZSPlus (Malvern Instruments; Malvern, UK). ZEN0040 cuvettes were used to measure the size, and DTS1070 cuvettes were used to measure the kinetic layer capability of the nanoparticles. All measurements were performed in triplicate at 25 °C. The percentage of encapsulated mRNA and mRNA concentrations were determined using the Quant-iT RiboGreen RNA reagent kit (Life Technologies, Waltham, MA, USA) according to the manufacturer’s protocol [43]. In brief, LNPs were diluted in either TE or TE mixed with Triton X-100 buffer (TX). Then, the Quant-iT RiboGreen assay quantified the unencapsulated mRNA (when diluted with TE) and total mRNA concentration (when diluted with TX). The amount of encapsulated mRNA was calculated by subtracting the unencapsulated mRNA concentration from the total mRNA concentration.

### 2.3. Laboratory Animals

Eight-week-old female and male BALB/c mice were obtained from the laboratory animal nursery of FBRI SRC Vector Rospotrebnadzor (Koltsovo, Russia). The mice were housed under a 12 h light/dark cycle with free access to food and water. Immunizations were administered under inhalation anesthesia (3% isoflurane solution) to minimize pain. Animal procedures, including infection and tissue sampling, were carried out following premedication with a combination of Zoletil 100 (Valdepharm, Val-de-Rey, France) and Xyla (Interchemie, Harju-Maakond, Estonia). All experiments adhered to the bioethical principles of the European Convention for the Protection of Vertebrate Animals Used for Experimental and Other Scientific Purposes (Strasbourg, 1986). The procedures involving animals were approved by the Bioethics Committee of SRC Vector (Protocol No. 2, 3 April 2023, approval code/02-03.2023).

### 2.4. Immunization of Mice

Four groups of female BALB/c mice (*n* = 5) were designated for histologic studies of tissue at the site of mRNA-GFP injection by jet injection. These included one group of intact control mice and three experimental groups (identified by protocol numbers). Mice in the experimental groups received a jet injection of mRNA-GFP at a dose of 30 μg in 50 μL saline.

Four groups of female BALB/c mice (*n* = 5) were designated for studies to test the functionality and stability of the mRNA-GFP molecule at the injection site by jet injection. These included one group of intact control mice and three experimental groups (identified by protocol numbers). Mice in the experimental groups received a jet injection of mRNA-GFP at a dose of 30 μg in 50 μL saline.

Three groups of female BALB/c mice (*n* = 5) were used to evaluate the distribution of Cy5-labeled mRNA-RBD (mRNA-RBD-Cy5) in vivo, either by jet injection or intramuscular injection with a needle and syringe. The first group received a jet injection (JET) of 30 μg mRNA-RBD-Cy5 in 50 μL saline, the second group was injected intramuscularly (I/M) with 30 μg mRNA-RBD-Cy5 in 50 μL saline using a needle and syringe, and the third group received 50 μL of PBS as a control. Mice were placed in an induction chamber and then under anesthesia masks. Hair was removed from the injection site on the leg with depilatory gel, the skin was treated with 70% ethanol, and mRNA-RBD-Cy5 was injected into the quadriceps muscle of the hind leg.

Four groups of female BALB/c mice (*n* = 16) were formed to study the immunogenic and protective properties of mRNA-RBD. The first group received an intramuscular (I/M) jet injection of 30 μg mRNA-RBD in 50 μL physiological solution (0.9% sodium chloride), the second group was injected I/M with 30 μg mRNA-RBD-LNP, the third group was injected I/M with 30 μg mRNA-RBD in 50 μL physiological solution, and the fourth group received an I/M injection of 50 μL physiological solution. Mice were immunized twice, with a three-week interval between doses. For each immunization, mice were placed in an induction chamber and then moved under anesthesia masks. Hair was removed from the injection site on the leg using a depilatory gel, and the skin was treated with 70% ethanol. The injection was administered into the quadriceps muscle of the hind leg. Ten days after the second immunization, blood samples were collected from six mice in each group to analyze the humoral immune response, and spleens were collected to assess the T-cell response. The remaining ten mice in each group were then infected with a live SARS-CoV-2 virus, strain hCoV-19/Russia/SA-17620-080521/2021, at a dose of 50 ID_50_.

### 2.5. Selection of Jet Injection Parameters for Optimal Conditions for mRNA Delivery In Vivo

To optimize the immunization protocol for mRNA delivery using the jet injection method, we used mRNA encoding GFP as a model (mRNA-GFP). Three protocols with varying flow pressures (3 to 10 bar), flow velocities (180 to 300 m/s), and injection times (0.22 to 0.44 s) were tested. Detailed information on the jet injection modes used is presented in Table 1. The experiment included four groups of animals: three experimental groups (designated by protocol numbers) and one intact control group, with ten mice in each group. mRNA-GFP was injected into the quadriceps femoris muscle of the left hind leg (100 μg in 50 μL saline per dose per mouse) using a Comfort-IN needle-free jet injector (MIKA MEDICAL CO., Busan, Republic of Korea).

Histological changes in muscle tissue at the injection site were analyzed following mRNA-GFP administration. GFP protein synthesis efficiency was assessed 48 h post-administration through microscopy of muscle tissue samples using an Olympus fluorescence microscope (Olympus Life Science, Tokyo, Japan). To quantify GFP fluorescence efficiency, image analysis software was used to measure the fluorescence intensity of GFP in muscle sections of BALB/c mice, as described previously [44].

### 2.6. Histological Studies

To evaluate the extent of tissue lesions, histological analysis was performed on mice at the site of mRNA-GFP vaccine injection, with samples collected 48 h after jet injection. Histological analysis was performed as previously described [44].

### 2.7. Visualization of Cy5-Tagged mRNA Distribution In Vivo

To visualize distribution, Cy5-tagged mRNA-RBD (30 μg in 50 μL) was used, with the Cy5 tag exhibiting an excitation peak at 651 nm and an emission peak at 670 nm. Cy5-tagged mRNA-RBD was administered either by a spring-loaded jet injector or by needle and syringe into the quadriceps muscle of the hind leg of BALB/c mice. Fluorescence activity was assessed qualitatively one-hour post-injection using an in vivo fluorescence and bioluminescence visualization and analysis system (VISQUE InVivo Series Smart-LF, Vieworks Co., Ltd., Anyang-si, Republic of Korea). Fluorescence analysis was conducted using CleVue^TM^ software 4.1.0.P 2616. 

### 2.8. ELISA

ELISA was conducted to assess the humoral immune response in BALB/c mice immunized with mRNA-RBD. Eukaryotic recombinant RBD protein, generously provided by the Laboratory of Immunogenetics, IMKB SB RAS, Novosibirsk, Russia, was used as the antigen. Proteins (1 μg/mL) were adsorbed in the wells of a 96-well polystyrene plate in PBS (Greiner Bio One GmbH, Frickenhausen, Germany). Mouse serum samples were added in triplicate serial dilutions starting at 1:50 and incubated at 37 °C for 60 min. After washing, rabbit anti-mouse IgG antibody conjugated to horseradish peroxidase (Sigma-Aldrich, St. Louis, MO, USA) and TMV substrate solution (Amresco LLC, Solon, OH, USA) were added. The optical density was measured at 450 nm on a Varioskan Lux instrument (Thermo Fisher Scientific, Waltham, MA, USA) after stopping the reaction [45].

### 2.9. SARS-CoV-2 Neutralization Assay

Vero E6 cells (epithelial cells from the kidney of the African green monkey) were used for the experiment, which were obtained from the Vector State Research Center collection (Rospotrebnadzor, Koltsovo, Russia). Cells were cultured in DMEM medium (Gibco, Thermo Fisher Scientific, Waltham, MA, USA) supplemented with L-glutamine, 10% fetal calf serum (Gibco, Thermo Fisher Scientific, Waltham, MA, USA), and antifungal antibiotic (Gibco, Thermo Fisher Scientific, Waltham, MA, USA) at 37 °C under 5% CO_2_.

The Coronavirus 2019-nCoV strain SARS-CoV-2/Australia/VIC01/2020 (from the State Collection of Viral Infectious Agents and Rickettsioses, SRC Vector, Rospotrebnadzor, Russia) was used for the study, which was conducted in a Biosafety Level 3 (BSL-3) laboratory. Neutralization assay was performed as previously described [45].

### 2.10. Obtaining Pseudoviruses and Neutralization Assays

To generate pseudoviral particles with SARS-CoV-2 S protein on their surface, HEK293 cell cultures were transfected with the plasmids psPAX2, pLV (IMBB SB RAS, Novosibirsk, Russia), and phS-Δ18 using Lipofectamine 3000 (Invitrogen, Waltham, MA, USA). The pseudoviral particles were collected by filtering the culture medium through a 0.45 μm filter followed by concentration in 20% sucrose solution. Aliquots of 500 μL were prepared and stored at −80 °C.

To assess the transducing activity of pseudoviral particles carrying the SARS-CoV-2 S glycoprotein and to conduct virus neutralization assays, 293-hACE2-TMPRSS2 target cells were used (Cell Culture Collection of FBUN RSC Vector, Rospotrebnadzor, Russia). This cell line stably expresses genes encoding hACE2 and TMPRSS2, with the corresponding receptors present on the cell surface. The transducing activity of pseudoviruses against target cells was determined according to the method described by Rudometova et al. [46]. Neutralization of pseudoviruses with immune sera was performed in 96-well culture plates following the protocol outlined by Neerukonda et al. [47].

### 2.11. IFN-γ ELISpot

The assay was conducted using the Abcam Murine IFNγ ELISPOT Kit (Abcam, Boston, MA, USA) with pre-coated plates following the manufacturer’s instructions. The quantity of IFN-γ-producing cells was determined using an ELISpot reader (Carl Zeiss, Oberkochen, Germany). To calculate the number of spot-forming cells per million cells, the mean value from the negative control wells was subtracted.

To evaluate IFN-gamma producing activity in the ELISpot test, splenocytes were placed in a well at a concentration of 3 × 10^5^/well. The cells were cultured in 5% CO_2_ at 37 °C for 20 h. Splenocytes isolated from immunized animals were stimulated with a pool of 13 peptides of RBD of SARS-CoV-2 (20 μg/mL of each peptide) recognized by major histocompatibility complex class I (H-2-Dd, H-2-Kd, H-2-Ld) and class II (H2-IAd, H2-IEd) molecules for BALB/c mice. Peptides were synthesized by AtaGenix Laboratories (Wuhan, China), with a peptide purity >80%.

### 2.12. Viral Challenge Experiment

The vaccine’s efficiency was evaluated against the gamma variant of SARS-CoV-2 (hCoV-19/Russia/SA-17620-080521/2021, GISAD ID: [EPI_ISL_6565014]) derived from a nasal swab specimen obtained from a patient in Yakutsk in May 2021. This strain was developed in Vero E6 cell culture, characterized, and deposited in the State Collection of Viral Infectious Agents and Rickettsioses at the FBRI SRC Vector Rospotrebnadzor (Koltsovo, Russia) [48]. The 50% infectious dose (ID_50_) of this strain for intranasal infection in BALB/c mice was previously determined in an experiment by Shipovalov et al. [49]. Four groups of BALB/c mice were infected with consecutive ten-fold dilutions of the viral efflux (with an efflux titer of no less than 10^6^ TCID_50_ /mL).

After 72 h, viral load was measured in lung and nasal cavity tissues by real-time RT-PCR and titration in the Vero E6 cell culture. The ID_50_ was calculated based on the proportion of vRNA-positive and vRNA-negative samples in each dilution group using the Reed and Muench formula. The ID_50_ was found to be 47.5 ± 1.8 for the strain hCoV-19/Russia/SA-17620-080521/2021 (gamma VOC) [49].

BALB/c mice were intranasally infected with a dose of 50 ID_50_ of SARS-CoV-2 virus, strain HCoV-19/Russia/SA-17620-080521/2021(gamma VOC), on day 31 after the first immunization.

The animals were euthanized on the fourth day post-infection. Tissue homogenates were prepared from specimens with an average weight of 0.1 g. Samples were homogenized in a FastPrep-24 homogenizer for 10 s at a speed of 5.5 m/s in chilled plastic tubes containing 1 mL of MEM medium at a ratio of 1:10 (mass: volume). Viral loads in the lungs and nasal cavities were evaluated using reverse transcription-quantitative polymerase chain reaction (RT-qPCR), as previously described by Dolskiy et al. (2020) [50]. RNA extraction was performed using the Riboprep kit (ILS, Moscow, Russia). The number of viral genome copies was determined using a diagnostic kit for SARS-CoV-2 (Vector Research Institute, Koltsovo, Russia) with a real-time PCR TaqMan assay. The Russian TaqMan PCR assay employed the following primer sequences:

5′-GTTGCAACTGAGGGAGCCTTG-3′ (forward), 5′-GAGAAGAGGCTTGACTGCCG-3′ (reverse), and 5′-FAM-TACACCAAAAGATCACATTGGCACCCG-BHQ1-3′ (internal).

The plasmid pJet1.2_SARS, which contains a fragment of the SARS-CoV-2 genome (strain MN997409.1, positions 28670-28826), was used as a control for quantitative PCR normalization. The quantity of viral genome copies was calculated using a DNA calculator (Thermo Fisher Scientific, Waltham, MA, USA) and the DNA concentration and dilution factors.

### 2.13. Statistical Analysis and Software

Data were analyzed using GraphPad Prism 9.0 software (GraphPad Software, Inc., San Diego, CA, USA). Results are presented as the median and range. Data were analyzed using the nonparametric Kruskal–Wallis test for one-factor analysis. Comparisons were not statistically significant unless otherwise stated.

## 3. Results

### 3.1. Selection and Optimization of Jet Injection Conditions

The parameters for jet injection were selected based on standard criteria—first, minimizing procedure-related trauma, and second, achieving stable biosynthesis of green fluorescent protein through mRNA-GFP translation. To this end, four groups of mice, each consisting of ten animals, were formed: three experimental groups and one control group (Table 1). mRNA-GFP was injected into the quadriceps femoris muscle of the left hind leg of mice (30 μg in 50 μL of physiological solution/dose/mouse) using a Comfort-IN jet injector (MIKA MEDICAL CO., Busan, Republic of Korea).

**Figure 1:**
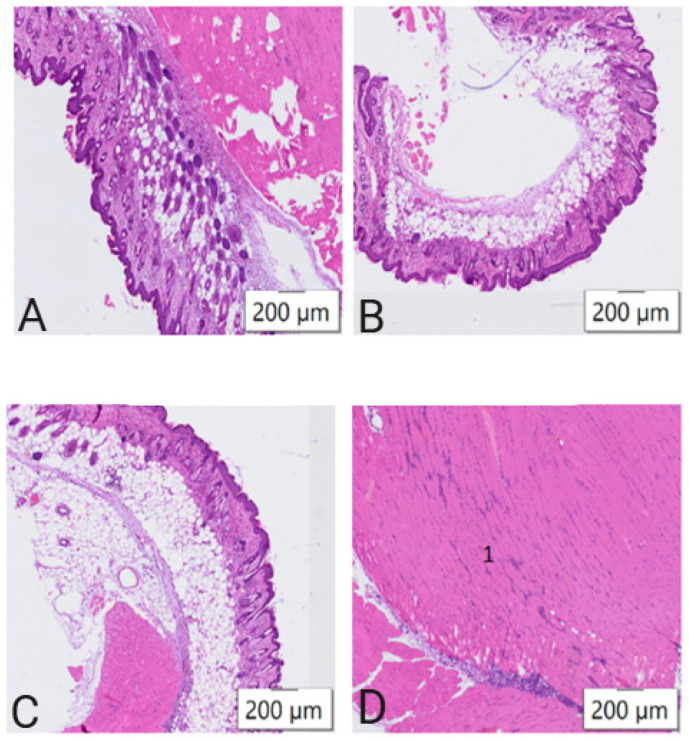
Microphotographs of tissue samples from mice showing histologic changes under different injection protocols (see Table 1). Samples taken from the injection site: (**A**) in control mice; (**B**) in mice to which protocol No. 1 was applied; (**C**) in mice to which protocol No. 2 was applied; (**D**) in mice to which protocol No. 3 was applied. Sections stained with hematoxylin and eosin. Denotations: 1, necrosis.

To assess tissue damage at the injection site, a histological examination of skin samples and adjacent muscle tissues was performed 48 h post-immunization. The degree of histopathologic changes varied according to the protocol used (Figure 1A–D). No differences from the control were observed with protocols No. 1 and No. 2. However, animals immunized following protocol No. 3 exhibited minor necrotic foci complications.

**Figure 2:**
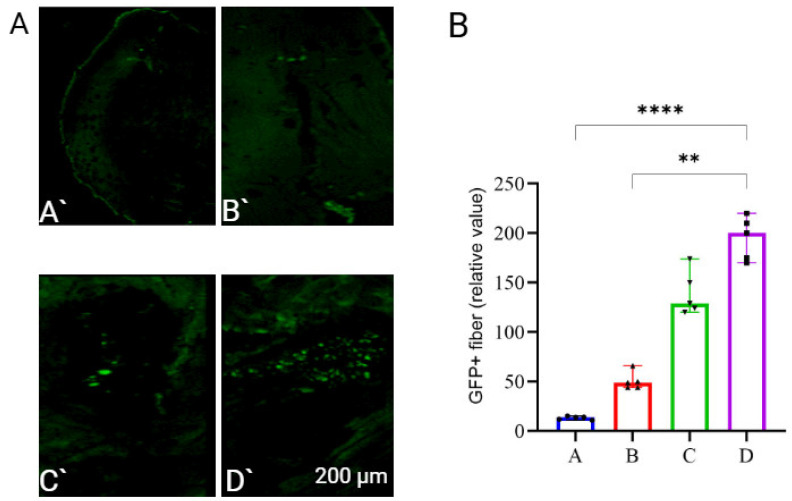
(**A**). Fluorescence microphotographs of muscle tissue obtained 3 days after immunization by jet injection of GFP plasmid. Samples were collected and then cut into 1 mm thick slices to visualize GFP expression: (**A’**) control mice (without jet injection); (**B’**) protocol No. 1; (**C’**) protocol No. 2; (**D’**) protocol No. 3. (**B**). Fluorescence intensity was plotted using computer-generated signal processing. (A) control mice (without jet injection); (B) protocol No. 1; (C) protocol No. 2; (D) protocol No. 3. Statistical significance was determined by nonparametric Kruskal–Wallis analysis with correction for multiple comparisons and Dunn’s test for statistical hypotheses (** *p* < 0.01, **** *p* < 0.0001).

GFP fluorescence was assessed by UV microscopy of thin muscle tissue sections from the animals on day 3 post-injection, coinciding with the onset of intense GFP fluorescence [44]. Representative micrographs are shown in Figure 2A.

Computer-assisted analysis quantified GFP fluorescence intensity in muscle slices from BALB/c mice. A conditional value for GFP+ fiber count was used, defined as the number of pixels within the green fluorescent spectrum relative to the entire image. This value was calculated by plotting a histogram of the image and marking the relative intensity distribution of pixel colors (Figure 2B).

Figure 2 shows that mice immunized using protocol No. 3 exhibited the highest GFP fluorescence intensity. The GFP signal intensity in this group was statistically significantly different from that of both the control group and the group immunized with protocol No. 2. However, due to the minor histologic damage associated with protocol No. 3, protocol No. 2 was chosen as the optimal method.

### 3.2. Distribution Naked mRNA-RBD-Cy5 Delivered by Intramuscular or Jet Injection

**Figure 3:**
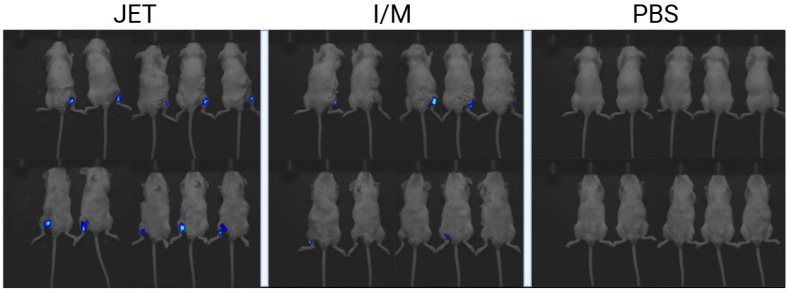
Visualization with in vivo imaging images showing the distribution of mRNA-RBD-Cy5 at the injection site, obtained using the VISQUE InVivo Series Smart-LF. mRNA-RBD-Cy5 was injected by jet injection into the quadriceps muscle of the left hind leg (JET) and intramuscularly (I/M) into the quadriceps muscle of the right hind leg of mice. Mice injected with PBS into the left hind leg were used as negative controls.

To evaluate the in vivo mRNA distribution, mRNA-RBD-Cy5 was administered via jet injection or by intramuscular injection with a needle and syringe, with PBS buffer used as a control. Results indicated that mRNA-RBD-Cy5 delivered via jet injection exhibited a wide distribution, high fluorescence intensity, and strict localization at the injection site (Figure 3). Compared to needle and syringe injection, jet injection showed a broader area and depth of mRNA-RBD-Cy5 distribution. This was further confirmed by fluorescence intensity measurements on the outer and inner surfaces of the animals’ legs.

These findings indicate that naked mRNA delivered by a spring-loaded jet injector achieves broad distribution at the injection site without causing systemic distribution throughout the organism.

### 3.3. Evaluation of the Immunogenic Properties of mRNA-RBD

**Figure 4:**
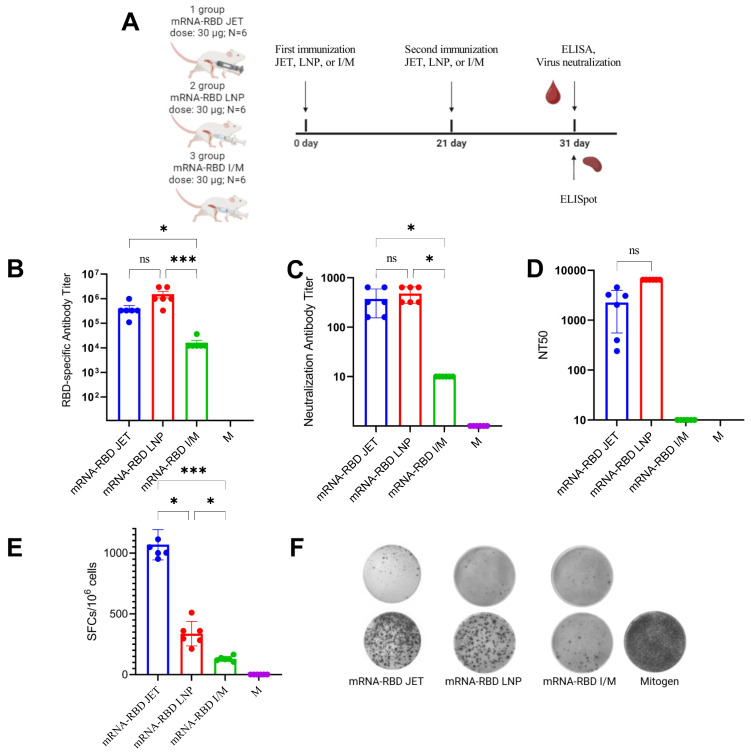
Study of the immunogenic properties of mRNA-RBD. (**A**) Immunization scheme. (**B**) Titers of specific IgG antibodies to RBD in sera of immunized animals (ELISA data). (**C**) Data from virus-neutralizing assay of sera using SARS-CoV-2 hCoV-19/Australia/VIC01/2020 strain (100 TCID_50_). (**D**) Data from virus-neutralizing serum assay using pseudotyped viruses carrying the S-glycoprotein of SARS-CoV hCoV-19/Australia/VIC01/2020 on their surface. (**E**) ELISpot assay results of specific T-cell responses in immunized BALB/c mice. Number of cells expressing IFN-γ in response to stimulation with a pool of RBD-specific peptides per 1 × 10^6^ splenocytes. (**F**) Representative images of ELISpot wells (top row: splenocytes not stimulated with peptides; bottom row: splenocytes stimulated with peptide pool or mitogen). In panels (**B**–**E**), data are presented as the median with a range of inverse titers. Significance was assessed using non-parametric one-factor Kruskal–Wallis analysis of variance (ns *p* > 0.05; * *p* < 0.05; *** *p* < 0.001). Designations: mRNA-RBD JET—mice immunized with naked RNA by jet injection, mRNA-RBD LNP—mice immunized with mRNA encapsulated in LNPs, mRNA-RBD I/M—mice immunized with naked RNA by intramuscularly, M—control (non-immunized) mice.

Synthesis of mRNA and encapsulation in LNPs were performed as described in the Materials and Methods. The average mRNA-RBD-LNP particle size was 145 nm, the polydispersity index was <0.15, the zeta potential was 0.23 mV, and the encapsulation efficiency was >98%. The immunization scheme for the animals is shown in Figure 4A. The humoral immune response to the mRNA-RBD vaccine was assessed by measuring the levels of RBD-specific antibodies and viral neutralizing activity in serum from immunized mice, which was collected on day 31 of the study. To evaluate RBD-specific antibody levels, sera from immunized animals were analyzed by ELISA. The mean RBD-specific antibody titer in group 1 mice (mRNA-RBD JET) was 1:400,967, and in group 2 mice (mRNA-RBD LNP), it was 1:1,530,922. However, the difference between these groups was not statistically significant (*p* = 0.213), indicating that both mRNA-RBD delivered via jet injection and mRNA-RBD encapsulated in LNPs and administered intramuscularly elicited comparable immune responses. The lowest antibody titer was observed in group 3 (mRNA-RBD I/M) at 1:16,200, which was 25-fold lower than that in group 1 (1:400,967).

To assess the virus-neutralizing activity of the serum, an in vitro virus neutralization assay was performed (Figure 4C) using two methods: a live virus (SARS-CoV-2 hCoV-19/Australia/VIC01/2020) and pseudotyped virus. We found that sera from mice immunized with mRNA-RBD-LNP neutralized the live SARS-CoV-2 hCoV-19/Australia/VIC01/2020 virus, achieving a mean neutralizing antibody titer of 1:17,066, which was 6.5-fold higher (*p* = 0.0514) than that of the mRNA-RBD JET group (titer 1:2560). Notably, neutralizing antibody levels in the mRNA-RBD JET group were more than 55-fold higher than those with intramuscular administration (titer 1:46, *p* < 0.0494). Similar results were obtained in the pseudotyped virus neutralization assay (Figure 4D), where sera from mice immunized with mRNA-RBD-LNP and mRNA-RBD-JET neutralized pseudotyped virus particles displaying SARS-CoV-2 S protein on their surface, with mean neutralizing antibody titers of 1:6480 and 1:2262, respectively (*p* = 0.7948).

The T-cell immune response in mice immunized with mRNA-RBD was evaluated using the ELISpot method, measuring the ability of splenocytes to secrete interferon-γ after specific stimulation with a pool of peptides from the RBD protein.

The results of the analyses presented in Figure 4E,F indicate significant differences in the level of cellular immune response in animals immunized by different methods. The highest number of IFN-γ-producing splenocytes in response to stimulation with RBD-specific peptides was observed in the group immunized via jet injection. This rate was 3-fold higher than that in the group of animals immunized with c/m mRNA-RBD-LNP (1068 vs. 337, *p* < 0.05) and 8-fold higher than that in the group immunized with c/m mRNA-RBD (1068 vs. 130, *p* < 0.05).

### 3.4. Evaluation of the Protective Efficiency of mRNA-RBD

**Figure 5:**
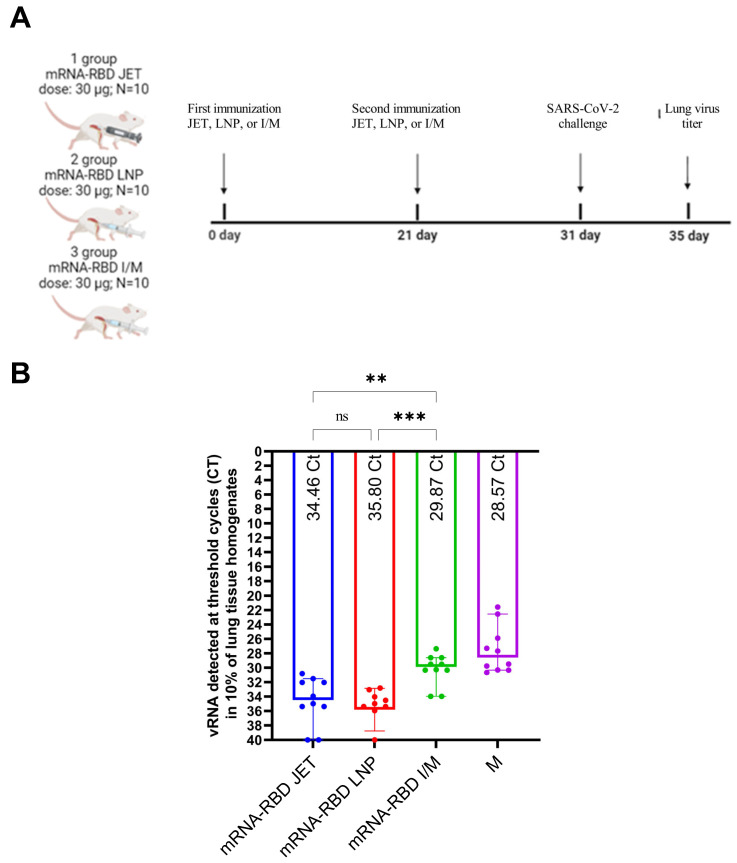
Examination of the protective activity of mRNA-RBD. (**A**) Immunization scheme. (**B**) Viral load in lung tissues of BALB/c mice on day 4 after infection with SARS-CoV-2 hCoV-19/Russia/SA-17620-080521/2021. Individual threshold cycle (Ct) values in real-time RT-qPCR are represented by dots; numerical values (Ct) are indicated at the base of the histograms. Statistical analysis was performed using the Kruskal–Wallis test (ns *p* > 0.05; ** *p* < 0.01; *** *p* < 0.001). Designations: mRNA-RBD JET—mice immunized with naked RNA by jet injection, mRNA-RBD LNP—mice immunized with mRNA encapsulated in LNP, mRNA-RBD I/M—mice immunized with naked RNA by intramuscularly, M—control (non-immunized) mice.

Next, the ability of mRNA-RBD to induce protective immunity against infection with the SARS-CoV-2 HCoV-19/Russia/SA-17620-080521/2021 (Gamma VOC) virus was evaluated on day 31 using a dose of 50 ID_50_. The Gamma variant of SARS-CoV-2 was chosen based on the susceptibility of BALB/c mice. Animals were euthanized on day 4 post-infection. Quantification of the viral load in 10% lung tissue homogenate was performed by real-time RT-qPCR with specific primers for the SARS-CoV-2 S gene. The results are presented in Figure 5 as threshold cycle (Ct) values.

The minimum viral load was detected in the group immunized with mRNA-RBD-LNP (median Ct value of 35.80). No statistically significant difference was observed between the mRNA-RBD-LNP and mRNA-RBD JET groups (median Ct 35.80 vs. 34.46, *p* = 0.3729). In the group immunized with mRNA-RBD I/M, the infectious virus titer in the lungs was reduced 10-fold compared to the experimental groups (*p* = 0.0045 for mRNA-RBD JET; *p* = 0.0002 for mRNA-RBD-LNP). In the control (non-immunized) animals, the threshold cycle for virus detection was 28.57, *p* < 0.0001.

## 4. Discussion

Although mRNA vaccines encapsulated in lipid nanoparticles (LNPs) have demonstrated a safety profile with minimal serious adverse events in clinical trials, there is opportunity to further reduce mRNA reactogenicity. The development of naked mRNA vaccines could improve vaccine tolerability. Naked nucleic acid delivery via jet injection could offer a solution.

The history of jet injectors began in the 1930s. In 1936, needleless systems were first described by Lockhart in his jet injection patent. Later, in the early 1940s, high-pressure devices were developed by Higson and others that used a fine jet of fluid to puncture the skin and inject drugs into underlying tissue [51,52]. These delivery systems allow rapid and painless vaccine administration, making them particularly appealing for mass vaccination efforts. Needle-free injection systems have been employed in vaccination campaigns to combat pandemics of infectious diseases such as smallpox, polio, influenza, and measles, as well as for delivering therapeutic drugs [22,53,54]. Although reusable injectors were widely used from the 1960s through the 1990s, they were found to contribute to cross-contamination, leading to their replacement by disposable syringes with needles in the 1990s [22,53,54,55,56]. Today, disposable injectors, which administer a single dose from a drug reservoir, are used for public vaccinations [54].

Jet injection as a method for nucleic acid delivery is a relatively recent development and was first tested with DNA vaccines. Jet injection has been shown to provide immunogenicity comparable to electroporation for DNA vaccines [26,42,43] and to elicit a stronger immune response than needle and syringe administration [53,57,58,59,60]. Importantly, the needle-free system may help reduce vaccine hesitancy due to needle phobia and lower the risk of accidental infections, such as viral hepatitis or HIV, among healthcare workers [53].

The use of jet injection for mRNA delivery is novel, and few studies have explored this method [19,61,62]. In these studies, both spring-loaded and pyro-driven injectors have been used. For example, Abbasi et al. employed a pyro-driven injector (PYRO-Jet) to deliver mRNA encoding the SARS-CoV-2 S-protein. They reported that injecting naked mRNA with the PYRO-Jet induced strong humoral and cellular immunity, with antigen expression predominantly localized at the injection site, which they believe is key to both improved vaccine efficacy and reduced systemic toxicity compared to mRNA-LNPs [61,62]. Abbasi et al. also noted that the PYRO injector induced localized proinflammatory responses at the injection site, presumably due to physical stress caused by the injection.

In our study, we used a spring injector for mRNA vaccine delivery, which differs from the PYRO injector in its method of generating injection pressure. Spring injectors, which operate based on kinetic energy generated by springs, are comparatively safer, simpler, more reliable, and easier to maintain. They also offer greater control and reproducibility in injections.

The first phase of our study focused on optimizing jet injection conditions using mRNA-GFP, which encodes green fluorescent protein, as a model molecule injected into BALB/c mice. Several evaluation criteria were applied to assess different jet injection protocols for mRNA vaccine delivery. A key criterion was the extent of tissue damage and inflammatory response compared to that with traditional syringe and needle injection. Histopathologic analysis served as the primary tool for evaluating the safety of this procedure.

We compared three jet injection protocols, with each differing in pressure, flow rate, and injection time for mRNA-GFP injection. The highest expression of mRNA-GFP, and thus the greatest GFP fluorescence at the injection site, was observed in mice immunized with protocol No. 3 parameters (Figure 2). However, protocol No. 3 led to minor necrotic foci in the animals. Protocol No. 2, which involved a right-angle injection at a pressure of 6.5 bar, a flow rate of 220 m/s, and an injection time of 0.33 s (Table 1), showed no tissue damage, as confirmed by histological results (Figure 1). With no observed histologic abnormalities, the GFP signal intensity in mice immunized using protocol No. 2 was not significantly different from that of protocol No. 3. Therefore, protocol No. 2 was selected for further study.

Following this, we evaluated the delivery efficiency of mRNA encoding the receptor-binding domain (RBD) of the SARS-CoV-2 S protein using a jet spring injector with protocol No. 2. Control groups received intramuscular injections of naked mRNA-RBD with a needle and syringe or mRNA-RBD encapsulated in LNPs. It is worth noting that the use of RNase inhibitors can enhance the efficiency and stability of naked RNA by forming complexes with RNases, thereby effectively preventing RNA degradation [63,64]. However, in our study, we only used naked mRNA constructs that did not contain RNase inhibitors.

ELISA results demonstrated that double immunization with mRNA-RBD using the optimized jet injection protocol induced a significantly stronger humoral immune response than that with intramuscular immunization. Notably, the titer of RBD-specific antibodies in the sera of mice immunized by jet injection was not significantly different from that of the group immunized with mRNA-RBD-LNP (Figure 4B).

The ability of mouse sera to inhibit viral infection in cells was tested in vitro using the hCoV-19/Australia/VIC01/2020 strain and Vero E6 cells. Antibody levels, including neutralizing antibodies, were found to be several times higher in the JET and LNP groups than in those immunized with the same doses of mRNA-RBD by traditional intramuscular injection (Figure 4). This effect is likely due to the increased delivery area and uniform distribution of mRNA in cells, enhancing overall antigen biosynthesis and, consequently, antibody production.

ELISpot analysis of cellular immune response showed that injection of naked mRNA induced a 3-fold increase in the number of specific IFN-γ-producing splenocytes compared with mRNA-RBD-LNP injection (Figure 4E,F). This may be because jet injection provides a more dispersed delivery of the mRNA vaccine, and as the skin serves as an immunologic barrier containing many antigen-presenting cells like Langerhans cells, it facilitates a high level of antigen presentation to immune cells, leading to a stronger T-cell response [65,66,67]. These results indicate the formation of a robust RBD-specific T-cell response.

Administration of both naked mRNA-RBD via jet injection and mRNA-RBD-LNP significantly reduced the viral load in mouse lungs compared to administration by syringe in the SARS-CoV-2 challenge experiment (Figure 5). Although mRNA-RBD-LNP was slightly more effective than jet injection, the difference was not statistically significant (Ct 35.80 vs. 34.46; *p* = 0.3729).

From a safety perspective, controlling mRNA distribution is critical for mRNA vaccine delivery systems. Multiple studies in both mice and nonhuman primates have shown that mRNA delivered by LNPs is detected in the inguinal and axillary lymph nodes, liver, and spleen [68,69,70,71,72,73], which enhances immunogenicity but may also increase reactogenicity. In contrast, mRNA delivered by jet injection remains localized at the injection site, supporting the safety of this administration method [61,62]. In our study, we monitored the distribution of naked mRNA-Cy5 after injection with a spring-loaded jet injector (Figure 3). Our data confirm that naked mRNA-RBD administered by this method has a broad distribution in tissue near the injection site but does not spread systemically while eliciting an immune response comparable to that with LNPs.

Thus, jet injection has advantages over LNPs. Jet injection enables high immunogenicity due to the broad tissue distribution and efficient cellular delivery of the drug. The high pressure generated by the jet injector may enhance mRNA internalization at the injection site without systemic distribution.

However, our study has a limitation, which is the use of a laboratory model of non-adapted BALB/c mice to study the effectiveness of protective properties using jet injection using the example of the mRNA-RBD mRNA construct encoding the receptor-binding domain of the SARS-CoV-2 spike protein. It is known that standard laboratory mice lack specific ACE2 receptors, which are necessary for SARS-CoV-2 to penetrate target cells, and, accordingly, effective infection of animals may not occur when studying the protective properties of the vaccine [74,75,76]. However, it was shown that BALB/c mice can be used as a model animal in screening studies when evaluating the effectiveness of therapeutic vaccine preparations and studying the solution pathogenesis caused by the VOC of SARS-CoV-2 viruses including Alpha (B.1.1.7), Beta (B.1.351), Gamma (P.1), Omicron (B.1.1.529) and the like in the study by Shipovalov et al., 2022 [49]. Thus, given the high values of antibody titers in virus neutralization and ELISA reactions, which were more than 55-fold higher than those with intramuscular administration, high protective activity can be expected when administering the mRNA vaccine by jet injection to transgenic mice and other experimental models. In the future, we plan to study the protective effect of mRNA delivered by the spring injector against more relevant SARS-CoV-2 strains.

## 5. Conclusions

In our study, we tested and confirmed the effectiveness of an inexpensive, simple, and safe method of mRNA delivery using a spring-loaded jet injector. Conditions were selected to ensure minimal tissue trauma and high expression of mRNA-GFP in the tissues of experimental animals. We demonstrated that naked mRNA-RBD delivered by jet injection resulted in the production of high titers of specific antibodies with virus-neutralizing activity, induced T-cell responses, and reduced the viral load in the lungs of mice following infection with SARS-CoV-2. The levels of humoral and T-cell immune responses in mice immunized with mRNA using the spring-loaded jet injector were comparable to those in animals injected with mRNA-RBD encapsulated in LNPs. Our findings suggest that jet injection may be a possible alternative to LNPs for delivering mRNA vaccines against SARS-CoV-2 infection.

## Figures and Tables

**Table 1:** Parameters of different jet injection protocols.

Number of the Protocol	1	2	3
Pressure (bar)	3	6.5	10
Flow speed (m/s)	180	220	300
Injection corner	90	90	90
Injection time (s)	0.22	0.33	0.44
Injection volume (µL)	50	50	50
Damage	No pathology	No pathology	Moderate level of infiltration
GFP fluorescence level (conventional units)	49	129	200

## Data Availability

The original contributions presented in this study are included in the article. Further inquiries can be directed to the corresponding author.
